# Static Electromechanical Characteristic of a Three-Layer Circular Piezoelectric Transducer

**DOI:** 10.3390/s20010222

**Published:** 2019-12-31

**Authors:** Grzegorz Mieczkowski, Andrzej Borawski, Dariusz Szpica

**Affiliations:** Bialystok University of Technology, Faculty of Mechanical Engineering, 45C Wiejska Str., 15-351 Bialystok, Poland; a.borawski@pb.edu.pl (A.B.); d.szpica@pb.edu.pl (D.S.)

**Keywords:** mechanical engineering, piezoelectric, electromechanical characteristic, circular piezoelectric transducer

## Abstract

The paper presents research related to the functional features of a novel three-layer circular piezoelectric actuator/sensor. The outer layers of the transducer are made of non-piezoelectric material. The middle layer comprises two elements—a piezoelectric disk, and a ring made of non-piezoelectric material. The additional external passive layer has a very important function; it protects the transducer’s electrical components against damage caused by external factors. Also, if sparking on the transducer wires or electrodes occurs, this layer prevents fire. So far, there is no analytical model for such a transducer. Closed-form analytical equations are important tools for predicting and optimizing the operation of devices. Thus, using both the Plate Theory and constitutive equations of piezoelectric materials, an analytical formula describing transducer deflection as a function of electrical loads has been found (electromechanical characteristic of the transducer). In addition, it is worth noting that under certain assumptions, the developed analytical model can also be used for two-layer transducers. The tests carried out show satisfactory compliance of the results obtained through the developed solution with both literature data and numerical data. Moreover, based on the obtained analytical model, the effect of selected non-dimensional variables on the actuator performance has been examined. These parameters include dimensions and mechanical properties of both piezoelectric disk and passive plates and strongly influence the behavior of the transducer.

## 1. Introduction

The first practical application of the piezoelectric phenomenon is attributed to Paul Langevin. In 1917, he developed the piezoelectric ultrasonic generator which was used to locate submarines. His invention started the increasingly frequent use of devices using the piezoelectric effect in many areas of the economy, such as medicine, industry, or transport. The principle of piezoelectric transducers performance, which may act as actuators [1,2,3] or sensors [4,5], is based on the conversion of electricity into mechanical energy, or vice versa. The relationship between the deformation of piezoelectric materials and an electric field is determined by the constitutive equations [6,7]. These equations form the basis for determining the electromechanical characteristics of piezoelectric actuators/sensors. On this basis, it is possible to predict transducer behavior and optimize its parameters. Such knowledge is very useful, because one can increase the converter efficiency at its design process. This can be achieved, for example, by obtaining higher transducer deflection when using less input power, often while reducing the transducer weight or dimensions. At present, before implementing and manufacturing a real device, the most common methods are numerical methods [8,9] or numerical-analytical methods [10,11]. However, many scientists, trying to provide more sophisticated design methods, develop new closed-form analytical solutions that are important tools for predicting and optimizing the operation of piezoelectric sensors/actuators.

The methods of obtaining analytical solutions and their form depend mainly on the geometrical and material features and conditions of mounting or loading the transducer. Most piezoelectric sensors/actuators usually have a layered structure, which causes some difficulties in determining their electromechanical characteristics. Interesting results of the analytical modeling of piezoelectric cantilever converters with equal length of all layers are presented in [12,13,14,15]. In [12], a double-layer transducer was analyzed. In the paper [13], there is the issue of a three-layer converter, whereas in [14,15], a multi-layer one. Static characteristics of double-layer converters in which piezoelectric layers have a different length than the passive ones are shown in [16,17]. To obtain the electromechanical characteristics of the transducers, the authors used the following methods: the elementary theory of elasticity [12], the energetic method [13,14], the Airy stress function method [15], the beam theory [16], the Lagrange method [17]. Analytical modeling of two- and three-layer transducers with freely-defined boundary conditions and geometry was also dealt with in [18,19,20]. To obtain electromechanical characteristics, the author [18,19,20] used the method based on the implementation of piezoelectric segments into the beam. A similar method was used by the authors of [21] for a two-layer circular piezoelectric converter. However, with double-layer circular transducers, the method based on Plate Theory is most often used to determine electromechanical characteristics [22,23,24,25]. This approach can also be used for three-layer [26] or multi-layer [27] circular piezoelectric converters. Additional layers (a piezoelectric or a passive one) in the transducer can perform various functions, e.g., they allow an increase in the deflection of the transducer while reducing its dimensions [28].

This article analyzes a novel three-layer circular piezoelectric transducer whose outer layers are made of non-piezoelectric material. The additional external passive layer protects the transmitter’s electrical components against damage caused by external factors. Furthermore, if there is sparking on the transducer wires or electrodes, this layer prevents fire. In the literature, analytical solutions describing the deflection of such three-layer circular piezoelectric transducers are difficult to find. Therefore, the main goal of this work is to develop procedures that allow the obtainment of the static electromechanical characteristics for such transducers.

The analytical solutions and the method of obtaining them are discussed in Section 2. It was also necessary to verify the correctness of the analytical model, as shown in Section 3. Section 4 presents the analysis of the impact of construction design on the deflection of three-layer circular piezoelectric transducers.

## 2. Electromechanical Characteristic of a Three-Layer Circular Piezoelectric Transducer

### 2.1. Basic Assumptions

**Figure 1:**
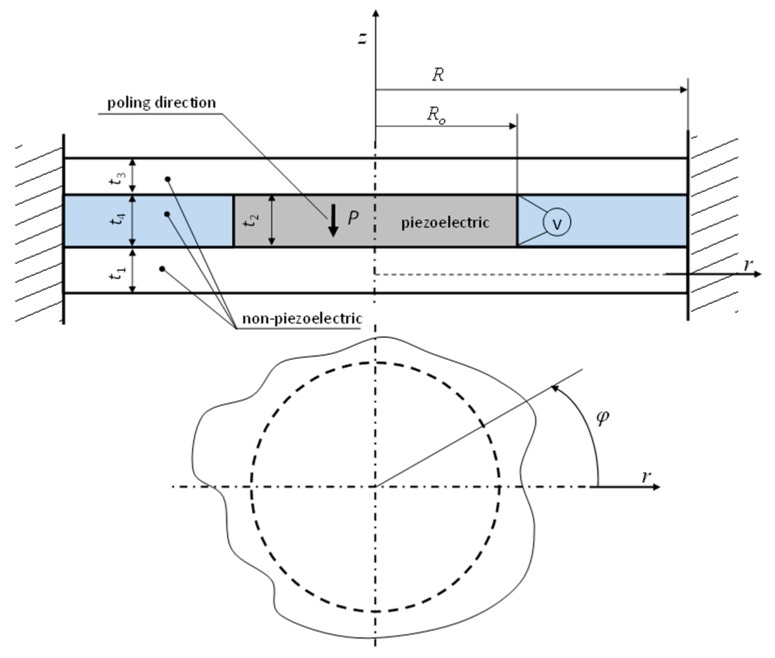
The structure schematic of the three-layer circular piezoelectric transducer.

The analyzed converter is made up of four different materials arranged in three layers (Figure 1). The outer layers, of thicknesses *t*_1_ and *t*_3_, are made of non-piezoelectric material. One of these layers is the executive element of the transducer, while the other one can act as a protective layer/coating. The middle layer consists of two components (of the same thickness *t*_2_ = *t*_4_)—a piezoelectric disk and a ring made of non-piezoelectric material. In this case, the material of the ring is foam. Its task is to stabilize the electric wires supplying current to the electrodes of the piezoelectric disk. The inner radius of the ring is equal to the outer radius of the piezoelectric disk and is denoted as *R_o_*. The outer radius of all layers is the same and equals *R*. All disks are fixed to and supported by outer cylindrical surfaces. The transducer deformation occurs because of the transverse piezoelectric effect (caused by the action of a *V* voltage) occurring inside the piezoelectric disk. In order to simplify the mathematical model, the following assumptions were made:

1. the total thickness of all layers is much smaller than their radius, therefore the Plate Theory [29] was used to determine transducer deflection;
2. the thickness of the adhesive layers and electrodes is very small and has no effect on the transducer deflection;
3. between individual transducer layers there are no slips and the cross-sections remain plane after deformation.
4. in the piezoelectric disk, only transverse piezoelectric effect occurs.

### 2.2. Analytical Description of Transducer Deformation

**Figure 2:**
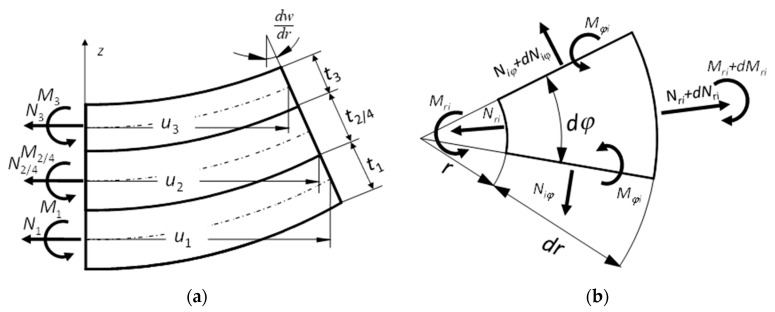
Fragment of the piezoelectric transducer structure: (**a**) deformed radial section, (**b**) system of forces acting on a small element of a transducer.

Figure 2 presents a fragment of the deformed structure of the analyzed transducer with internal forces occurring in individual layers. 

Based on the small deflection theory of round thin axisymmetric plate [28], and assuming that the transducer is not subject to any external loads, equations of forces and bending moments (Figure 2b) can be written as follows:

(1) $$\frac{dN_{r}}{dr}+\frac{N_{r}-N_{\varphi }}{r}=0,\;\frac{dM_{r}}{dr}+\frac{M_{r}-M_{\varphi }}{r}=0.$$

where, *N_r_*, *M_r_*—total force and bending moment in the radial direction; *N_φ_*, *M_φ_*—total force and bending moment in the circumferential direction.

It is worth noting that the above equation is valid only on the assumption that shear deformation and rotary inertia can be omitted [29]. Therefore, in order to be able to use equation 1, the deformation of the piezoelectric disk caused by the longitudinal and shear piezoelectric effects has been not taken into account in this paper. Such simplification is used by many researchers, for example by the authors of the papers [21,24,25].

For the deflected transducer, the radial and circumferential strain–displacement relationships are as follows:

(2) $$\varepsilon_{ri}=\frac{du_{i}}{dr}-z\frac{d^{2}w}{dr^{2}},\;\varepsilon_{\varphi i}=\frac{u_{i}}{r}-z\frac{1}{r}\frac{dw}{dr},\;i=1,2,3,4.$$

Assuming that the cross-sections of individual layers remain flat after deformation (Figure 2a), the displacement of the individual layers can be made dependent on the displacement of the lower layer 1:

for the interval *r* < *R_o_*:
  (3) $$u_{2}=u_{1}-\frac{t_{1}+t_{2}}{2}\frac{dw}{dr},\;u_{3}=u_{1}-\frac{t_{1}+t_{3}+2t_{2}}{2}\frac{dw}{dr}$$
for the interval *R_o_* < *r* < *R*
  (4) $$u_{4}=u_{1}-\frac{t_{1}+t_{4}}{2}\frac{dw}{dr},\;u_{3}=u_{1}-\frac{t_{1}+t_{3}+2t_{4}}{2}\frac{dw}{dr}.$$

Applying Hook’s law, and taking into account the transverse piezoelectric effect in a piezoelectric disk, radial $\sigma_{ri}$ and circumferential $\sigma_{\varphi i}$ stresses occurring in individual layers of the transducer can be written using the following equations:

for the interval *r* < *R_o_*:
  (5) $$\begin{array}{l} \sigma_{r1}=\frac{E_{1}\left(\varepsilon_{\varphi }{}_{1}\nu_{1}+\varepsilon_{r1}\right)}{1-\nu_{1}{}^{2}},\;\sigma_{\varphi 1}=\frac{E_{1}\left(\varepsilon_{\varphi }{}_{1}+\varepsilon_{r1}\nu_{1}\right)}{1-\nu_{1}{}^{2}}\\ \sigma_{r2}=\frac{E_{2}\left(\varepsilon_{\varphi }{}_{2}\nu_{2}+\varepsilon_{r2}+d_{31}\frac{V}{t_{2}}\left(1+\nu_{2}\right)\right)}{1-\nu_{2}{}^{2}},\;\sigma_{\varphi 2}=\frac{E_{2}\left(\varepsilon_{\varphi }{}_{2}+\varepsilon_{r2}\nu_{2}+d_{31}\frac{V}{t_{2}}\left(1+\nu_{2}\right)\right)}{1-\nu_{2}{}^{2}}\\ \sigma_{r3}=\frac{E_{3}\left(\varepsilon_{\varphi }{}_{3}\nu_{3}+\varepsilon_{r3}\right)}{1-\nu_{3}{}^{2}},\;\sigma_{\varphi 3}=\frac{E_{3}\left(\varepsilon_{\varphi }{}_{3}+\varepsilon_{r3}\nu_{3}\right)}{1-\nu_{3}{}^{2}} \end{array}\}$$
for the interval *R_o_* < *r* < *R*
  (6) $$\begin{array}{l} \sigma_{r1}=\frac{E_{1}\left(\varepsilon_{\varphi }{}_{1}\nu_{1}+\varepsilon_{r1}\right)}{1-\nu_{1}{}^{2}},\;\sigma_{\varphi 1}=\frac{E_{1}\left(\varepsilon_{\varphi }{}_{1}+\varepsilon_{r1}\nu_{1}\right)}{1-\nu_{1}{}^{2}}\\ \sigma_{r4}=\frac{E_{4}\left(\varepsilon_{\varphi }{}_{4}\nu_{4}+\varepsilon_{r4}\right)}{1-\nu_{4}{}^{2}},\;\sigma_{\varphi 4}=\frac{E_{4}\left(\varepsilon_{\varphi }{}_{4}+\varepsilon_{r4}\nu_{4}\right)}{1-\nu_{4}{}^{2}}\\ \sigma_{r3}=\frac{E_{3}\left(\varepsilon_{\varphi }{}_{3}\nu_{3}+\varepsilon_{r3}\right)}{1-\nu_{3}{}^{2}},\;\sigma_{\varphi 3}=\frac{E_{3}\left(\varepsilon_{\varphi }{}_{3}+\varepsilon_{r3}\nu_{3}\right)}{1-\nu_{3}{}^{2}} \end{array}\}$$

where, *E_i_*—Young’s modules; *ν_i_*—Poisson’s ratios; *d*_31_—piezoelectric constant.

Forces and bending moments occurring in individual transducer layers are determined on the basis of Equations (2)–(6):

for the interval *r* < *R_o_*:
  (7) $$N_{r1}=\int_{-t_{1}/2}^{t_{1}/2}\sigma_{r1}dz,N_{r2}=\int_{-t_{2}/2}^{t_{2}/2}\sigma_{r2}dz,N_{r3}=\int_{-t_{3}/2}^{t_{3}/2}\sigma_{r3}dz$$
  (8) $$N_{\varphi 1}=\int_{-t_{1}/2}^{t_{1}/2}\sigma_{\varphi 1}dz,N_{\varphi 2}=\int_{-t_{2}/2}^{t_{2}/2}\sigma_{\varphi 2}dz,N_{\varphi 3}=\int_{-t_{3}/2}^{t_{3}/2}\sigma_{\varphi 3}dz$$
  (9) $$M_{r1}=\int_{-t_{1}/2}^{t_{1}/2}\left(\sigma_{r1}z\right)dz,M_{r2}=\int_{-t_{2}/2}^{t_{2}/2}\left(\sigma_{r2}z\right)dz,M_{r3}=\int_{-t_{3}/2}^{t_{3}/2}\left(\sigma_{r3}z\right)dz$$
  (10) $$M_{\varphi 1}=\int_{-t_{1}/2}^{t_{1}/2}\left(\sigma_{\varphi 1}z\right)dz,M_{\varphi 2}=\int_{-t_{2}/2}^{t_{2}/2}\left(\sigma_{\varphi 2}z\right)dz,M_{\varphi 3}=\int_{-t_{3}/2}^{t_{3}/2}\left(\sigma_{\varphi 3}z\right)dz$$
for the interval *R_o_* < *r* < *R*
  (11) $$N_{r1}=\int_{-t_{1}/2}^{t_{1}/2}\sigma_{r1}dz,N_{r4}=\int_{-t_{4}/2}^{t_{4}/2}\sigma_{r4}dz,N_{r3}=\int_{-t_{3}/2}^{t_{3}/2}\sigma_{r3}dz$$
  (12) $$N_{\varphi 1}=\int_{-t_{1}/2}^{t_{1}/2}\sigma_{\varphi 1}dz,N_{\varphi 4}=\int_{-t_{4}/2}^{t_{4}/2}\sigma_{\varphi 4}dz,N_{\varphi 3}=\int_{-t_{3}/2}^{t_{3}/2}\sigma_{\varphi 3}dz$$
  (13) $$M_{r1}=\int_{-t_{1}/2}^{t_{1}/2}\left(\sigma_{r1}z\right)dz,M_{r4}=\int_{-t_{4}/2}^{t_{4}/2}\left(\sigma_{r4}z\right)dz,M_{r3}=\int_{-t_{3}/2}^{t_{3}/2}\left(\sigma_{r3}z\right)dz$$
  (14) $$M_{\varphi 1}=\int_{-t_{1}/2}^{t_{1}/2}\left(\sigma_{\varphi 1}z\right)dz,M_{\varphi 2}=\int_{-t_{2}/2}^{t_{2}/2}\left(\sigma_{\varphi 2}z\right)dz,M_{\varphi 3}=\int_{-t_{3}/2}^{t_{3}/2}\left(\sigma_{\varphi 3}z\right)dz$$

Resultant force and bending moment are the sum of forces and bending moments occurring in individual layers:

(15) $$N_{r}=\sum_{i}N_{ri},N_{\varphi }=\sum_{i}N_{\varphi i},M_{r}=\sum_{i}M_{ri},M_{\varphi }=\sum_{i}M_{\varphi i}$$

where *i* = 1, 2, 3 for the interval *r* < *R_o_*; *i* = 1, 2, 4 for the interval *R_o_* < *r* < *R*.

Using Equations (15) and (1), a differential equation of displacement of the lower layer of the transducer (16) and its deflection (17) is obtained:

(16) $$\frac{d^{2}u_{1j}(r)}{dr^{2}}+\frac{du_{1j}(r)}{r\;dr}-\frac{u_{1j}(r)}{r^{2}}=0,$$

(17) $$\frac{d^{3}w_{j}(r)}{dr^{3}}+\frac{d^{2}w_{j}(r)}{r\;dr^{2}}-\frac{dw_{j}(r)}{r^{2}\;dr}=0.,$$

where *j* = *I* for the interval *r* < *R_o_*; *j* = *II* for the interval *R_o_* < *r* < *R*.

The general solution of the above differential equations has the following form:

(18) $$u_{1j}(r)=\frac{D_{1j}r}{2}+\frac{D_{2j}}{r},w_{j}(r)=\frac{C_{1j}r^{2}}{4}+C_{2j}\ln\left(r\right)+C_{3j}$$

The integration constants are determined from the following boundary conditions (19) (assuming that the transducer midpoint deflection is limited) and the continuity conditions of the corresponding fields for *r* = *R_o_* (20):

(19) $$u_{1I}(0)<\infty ;\;\frac{dw_{I}(r)}{dr}|{}_{r=0}<\infty ;\;\frac{dw_{II}(r)}{dr}|{}_{r=R}=0;\;w_{II}(R)=0;\;u_{1II}(R)=0,$$

(20) $$\begin{array}{l} \frac{dw_{I}(r)}{dr}|{}_{r=R_{o}}=\frac{dw_{II}(r)}{dr}|{}_{r=R_{o}};\;w_{I}(R_{o})=w_{II}(R_{o});u_{1I}(R_{o})=u_{1II}(R_{o})\\ N_{r1I}(R_{o})=N_{r1II}(R_{o});M_{rI}(R_{o})=M_{rII}(R_{o}) \end{array}\},$$

where $N_{r1I}(R_{o})$ and $N_{r1II}(R_{o})$ are calculated from Equations (7) and (11). The bending moments in layer 1 are calculated from the below equations:

for the interval *r* < *R_o_*:
  (21) $$M_{rI}(R_{o})=M_{r1}(R_{o})+M_{r2}(R_{o})+M_{r3}(R_{o})+N_{r3}(R_{o})\frac{\left(t_{1}+t_{3}+2t_{2}\right)}{2}+N_{r2}(R_{o})\frac{\left(t_{1}+t_{2}\right)}{2}$$
for the interval *R_o_* < *r* < *R*:
  (22) $$M_{rII}(R_{o})=M_{r1}(R_{o})+M_{r4}(R_{o})+M_{r3}(R_{o})+N_{r3}(R_{o})\frac{\left(t_{1}+t_{3}+2t_{4}\right)}{2}+N_{r4}(R_{o})\frac{\left(t_{1}+t_{4}\right)}{2},$$

where *M_ri_*(*R_o_*) and *N_ri_*(*R_o_*) are determined using the Equations (7) and (9) (in Formula (21)), and Equations (11) and (13) (in Formula (22)).

Using the above conditions and the Formula (18), the equation describing the deflection of the transducer was obtained:

(23) $$w(r)=\left\{ \begin{array}{l} w_{I}(r)=\frac{A_{I}}{C},r\leq R_{o}\\ w_{II}(r)=\frac{A_{II}}{C},R_{o}\leq r\leq R \end{array}\right.,$$

where:

$$\begin{array}{l} A_{I}=-3d_{31}E_{2}VR_{o}{}^{2}\left(t_{1}+t_{2}\right){\left(1-\nu_{1}{}^{2}\right)}^{2}\left(1+\nu_{2}\right)\left(r^{2}\left(1-\frac{R^{2}}{R_{o}{}^{2}}\right)+2R^{2}\ln\left(\frac{R}{R_{o}}\right)\right),\\ A_{II}=-3d_{31}E_{2}VR_{o}{}^{2}\left(t_{1}+t_{2}\right){\left(1-\nu_{1}{}^{2}\right)}^{2}\left(1+\nu_{2}\right)\left(\left(r^{2}-R^{2}\right)+2R^{2}\ln\left(\frac{R}{r}\right)\right),\\ C=\left(1-\nu_{1}{}^{2}\right)\left(\nu_{2}{}^{2}-1\right)\left(2E_{1}R^{2}t_{1}{}^{3}+\frac{\left(\nu_{1}{}^{2}-1\right)\left(F_{I}+\left(-1+\nu_{2}\right)\left(2E_{3}t_{3}\left(F_{IV}+F_{II}\right)\left(-1+\nu_{4}{}^{2}\right)+F_{III}\right)\right)}{\left(\nu_{2}-1\right)\left(\nu_{3}{}^{2}-1\right)\left(\nu_{4}{}^{2}-1\right)}\right),\\ F_{I}=E_{2}\left(\nu_{3}{}^{2}-1\right)\left(\nu_{4}{}^{2}-1\right)\left(R^{2}-Ro^{2}\right)\left(3t_{1}{}^{2}+6t_{1}t_{2}+4t_{2}{}^{2}\right)t_{2},\\ F_{II}=R^{2}\left(3t_{1}{}^{2}+6t_{1}t_{2}+6t_{2}{}^{2}+6t_{1}t_{3}+6t_{2}t_{3}+4t_{3}{}^{2}+6t_{1}t_{4}+6t_{3}t_{4}+6t_{4}{}^{2}+6\left(t_{2}-t_{4}\right)\left(t_{1}+t_{2}+t_{3}+t_{4}\right)\nu_{3}\right),\\ F_{III}=E_{4}t_{4}\left(3t_{1}{}^{2}+6t_{1}t_{4}+4t_{4}{}^{2}\right)\left(\nu_{3}{}^{2}-1\right)\left(R_{o}{}^{2}\left(\nu_{4}+1\right)-R^{2}\left(\nu_{4}-1\right)\right),\\ F_{IV}=-6R_{o}{}^{2}\left(t_{2}-t_{4}\right)\left(t_{1}+t_{2}+t_{3}+t_{4}\right)\left(1+\nu_{3}\right). \end{array}$$

## 3. Verification of the Analytical Solution

**Figure 3:**
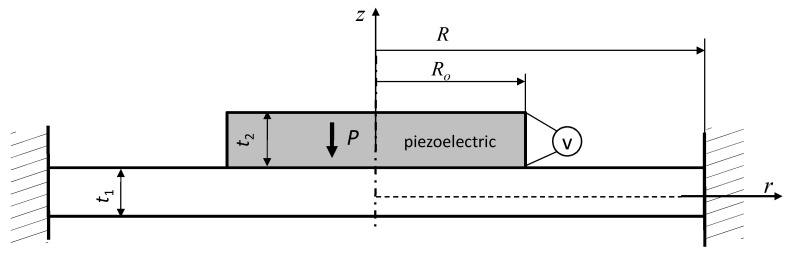
The unimorph actuator.

To check the correctness of the obtained analytical solution, electromechanical characteristics were developed for transducers with specific geometrical-material parameters and the obtained results were compared with both literature data and the results obtained by the Finite Element Method (FEM). Because no information was found in the literature for the exact transducer analyzed in the present work, based on the analytical solution (23), the characteristics for the transducer shown in Figure 3 were developed. The solutions for the transducer shown in the figure below were obtained by adopting (in Equation (23)) the following assumptions: *E*_3_ = *E*_4_ = 0, *t*_3_ = *t*_4_ = 0. 

**Figure 4:**
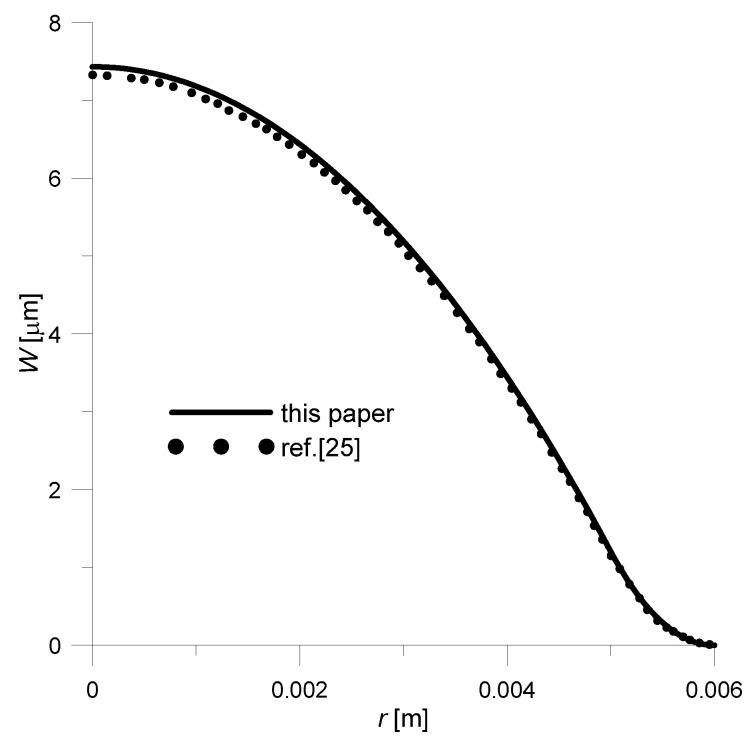
Comparison of deflection curves: solid line—analytical solution (23); dashed line—analytical solution taken from the work [25] (p. 36), *V* = 200 V, *R* = 6 × 10^−3^ m, *R_o_* = 5 × 10^−3^ m, *t*_1_ = 2 × 10^−4^ m, *t*_2_ = 1.5 × 10^−4^ m, Pz26/Cu.

The obtained solution had to be compared to the ones available in the literature. Therefore, assuming—as the authors of the work did [25]—that the piezoelectric disk is made of Pz26, and the lower layer made of copper, a deflection curve was prepared (Figure 4). As for geometrical dimensions, they were respectively: *R* = 6 × 10^−3^ m, *R_o_* = 5 × 10^−3^ m, *t*_1_ = 2 × 10^−4^ m, *t*_2_ = 1.5 × 10^−4^ m. In addition, the driving voltage was set to 200 V.

As there was no specific material data for Pz26 and Cu given by authors of paper [25], it was taken from [29]: *E*_2_ = 1/*s*_11_ = 7.69 × 10^10^ Pa, ν_2_ = −*s*_12_/*s*_11_ = 0.334, *d*_31_ = −1.3 × 10^−10^ m/V, *E*_1_ = 13 × 10^10^ Pa, *ν*_1_ = 0.34. Comparing the results obtained with the literature data (Figure 4), it can be concluded that both solutions are approximately equal—the maximum error is 1.3%. This slight difference in results could be due to the fact that comparative data (dashed line, Figure 4) was obtained by digitizing the graph from [25].

**Figure 5:**
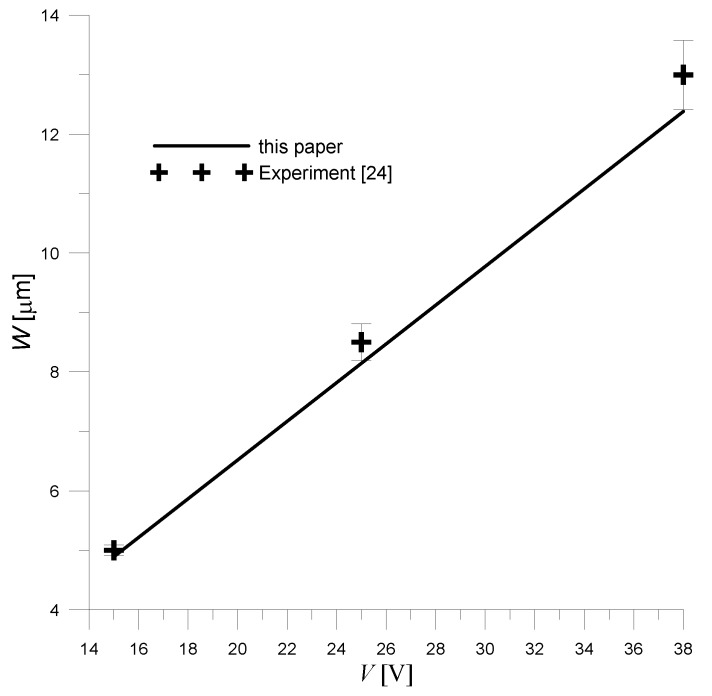
Comparison of the analytical solution (23) obtained for the unimorph actuator with experimental data [24] (Adapted from Mo, C.; Wright, R.; Slaughter, W.S.; Clark, W.W Behaviour of a unimorph circular piezoelectric actuator. Smart Mater. Struct. 2006, 15, p. 1102).

The analytical solution obtained had to be verified experimentally too. Therefore, the analytical solution obtained for the unimorph actuator (Figure 3) was compared with the experimental one. Assuming, just like the authors of [24], the following:

(a) geometrical dimensions: *R* = 2.54 × 10^−2^ m, *R_o_* = 1.27 × 10^−2^ m, *t*_1_ = 5.08 × 10^−4^ m, *t*_2_ = 1.127 × 10^−4^ m
(b) material data:
  - bottom layer made of aluminium; *E*_1_ = 70 × 10^9^ Pa, *ν*_1_ = 0.33;
  - piezoelectric disk made of PZT-5H (Piezo Material Lead Zirconate Titanate); *E*_2_ = 1/*s*_11_ = 6.06 × 10^10^ Pa, *ν*_2_ = −*s*_12_/*s*_11_ = 0.289, *d*_31_ = −2.74 × 10^−10^ m/V;

The transducer center point deflection (*r* = 0) was determined. Furthermore, the deflection was determined for different values of driving voltages. The obtained results are presented in Figure 5.

Analyzing the obtained results (Figure 5), it can be stated that the analytical solution agrees with the experimental ones. However, it can be seen that as the applied voltage increases, a greater discrepancy between theoretical and experimental results is obtained. This may be due to the fact that it is difficult to achieve a true clamped condition on the outer walls of the real transducer (this is reported by the authors of experimental studies [24]). The impact of potential support flexibility on the electromechanical characteristics of the transducer increases as its deflection increases. The deflection is proportional to the applied voltage, so as the voltage increases, the difference between the results obtained from the theoretical equations and the experiment increases.

As already mentioned, another way of verification was to compare an analytical solution with a numerical one. The Finite Element Method is one of the most frequently used numerical methods. Currently, FEM is used in virtually all fields of science. FEM analyses can be performed for any load conditions (static, dynamic) and take into account (together with experimental research) frictional and flow aspects [30,31,32,33,34,35].

Therefore, using the finite element method, the analyzed three-layer transducer (Figure 1) was modeled and the obtained electromechanical characteristics were compared with the analytical solution (23). The deflection of the converter was simulated using the COMSOL Multiphysics (Comsol Multiphysics GmbH, Berlin, Germany) software [36,37,38]. A half symmetry axisymmetric model of the transducer was built using six-node triangular plane elements. A self-adaptive finite element mesh was used, matching the modeled geometry and physical phenomenon (in the COMSOL environment this option is called “Physics-controlled mesh”). In addition, the mesh was manually condensed at the end of both the piezoelectric disk and the transducer. It is worth mentioning that when modeling piezoelectric materials using COMSOL software, there is no need to manually choose a specific type of element (e.g., as it is in the ANSYS Mechanical APDL environment). The finite element type is selected automatically depending on the modeled physical problem. As for the boundary conditions (support conditions, electrical load), they have been modeled in a way corresponding to the actual operating conditions of the transducer (described in Section 2.1).

When analyzing, the following assumptions were made:

(a) geometrical dimensions: *R* = 6 × 10^−2^ m, *R_o_* = 5.5 × 10^−2^ m, *t*_1_ = 2.5 × 10^−4^ m, *t*_2_ = *t*_4_ = 2.5 × 10^−4^ m, *t*_3_ = 3 × 10^−4^ m.
(b) material data:
  - bottom layer made of copper; *E*_1_= 13 × 10^10^ Pa, *ν*_1_ = 0.34;
  - upper layer made of PTFE (Polytetrafluoroethylene); *E*_3_ = 0.4 × 10^9^ Pa, *ν*_3_ = 0.46;
  - piezoelectric disk made of PZT-5H (Piezo Material Lead Zirconate Titanate); *E*_2_ = 1/*s*_11_ = 6.06 × 10^10^ Pa, *ν*_2_ = −*s*_12_/*s*_11_ = 0.289, *d*_31_ = −2.74 × 10^−10^ m/V;
  - the middle ring made of foam; *E*_4_ = 35.8 × 10^6^ Pa, *ν*_4_ = 0.383.
(c) applied electrical load: *V* = −100 V.

**Figure 6:**
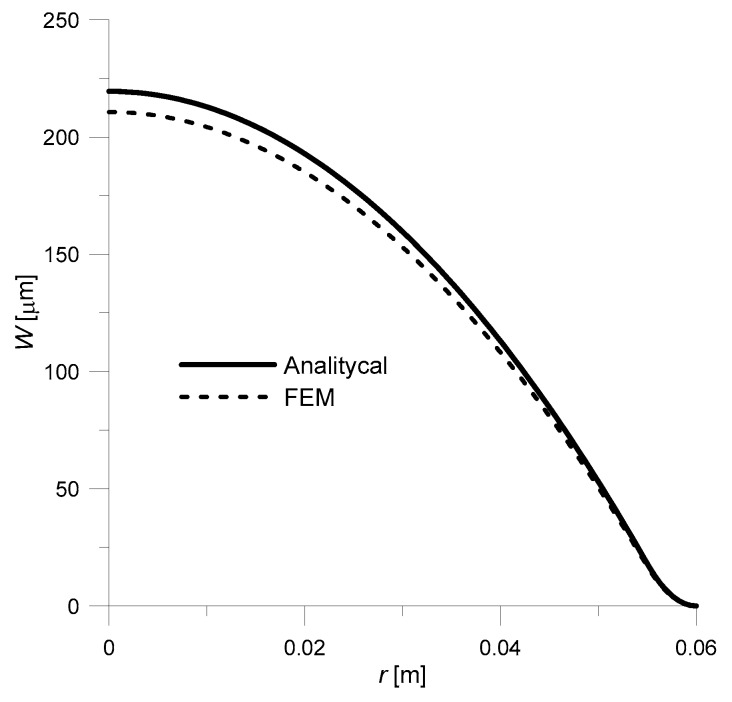
Comparison of deflection curves for the three-layer transducer: solid line—analytical solution (20); dashed line—FEM (Finite Element Method) solution; *V* = −100 V.

The obtained results are presented in Figure 6.

Based on the obtained results, it can be concluded that both solutions are consistent—the maximum lapse was 4.1%. In the solution obtained by FEM, slightly lower deflection values are obtained (this fact was also noted in [25]), which may be due to the fact that the analytical solution assumes that the cross-section after deformation remains flat.

## 4. Influence of Geometrical-Material Parameters on the Electromechanical Characteristics of a Three-Layer Transducer 

The performed analyses allowed the determination of the influence of the geometrical and material parameters (represented as non-dimensional variables) of individual transducer components on its functional features. The analyses were carried out in two steps. First, the transducer was treated as a global structure composed of piezo and non-piezoelectric materials. The impact on transducer deflection of non-dimensional variables such as,

- the relative thickness of piezo and non-piezo elements: *t_g_* = *t*_2_/(*t*_1_ + *t*_3_);
- the elastic moduli ratio of piezo and non-piezoelectric components: *E_g_* = *E*_2_/(*E*_1_ + *E*_3_);
- the relative radius of piezoelectric disk and non-piezoelectric layers: *R_g_* = *R_o_*/*R*;

were investigated.

**Figure 7:**
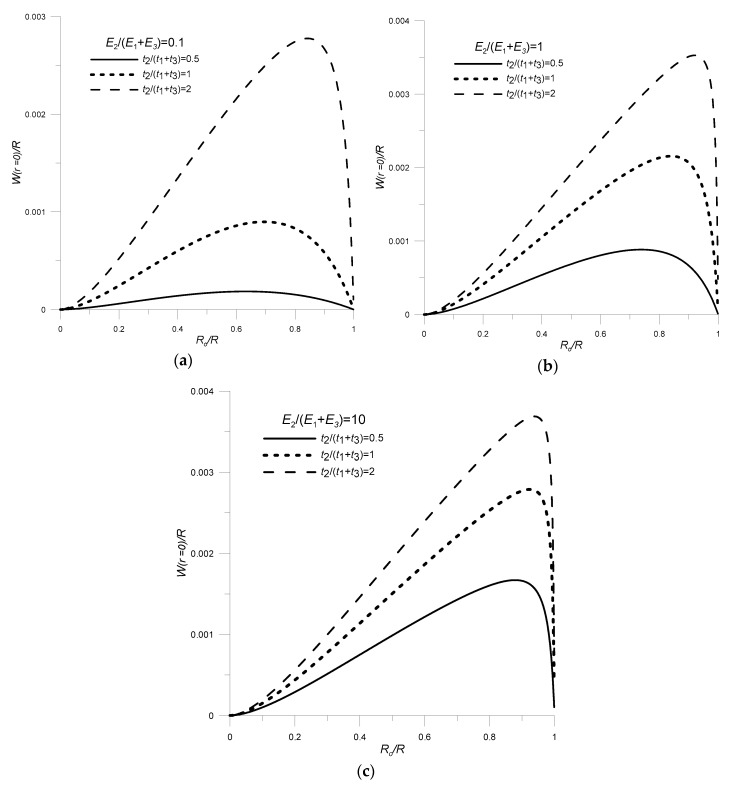
Influence of the ratio of elastic moduli, the relative thickness and the radius of piezo and non-piezoelectric materials on transducer deflection: (**a**) *E_g_* = 0.1, (**b**) *E_g_* = 1, (**c**) *E_g_* = 10; *ν*_1_ = 0.34, *ν*_3_ = 0.46, *R* = 0.06 m, *t*_3_ = 0.0001 m, *V* = 100 V.

These results are graphically represented in Figure 7.

Then the influence of mutual geometrical and material relations of non-piezoelectric layers on deformation conditions was analyzed. The following factors were considered:

- the relative thickness of the top and bottom layers: *t_np_* = *t*_3_/*t*_1_;
- the ratio of elastic moduli of non-electrical layers: *E_np_* = *E*_3_/*E*_1_.

**Figure 8:**
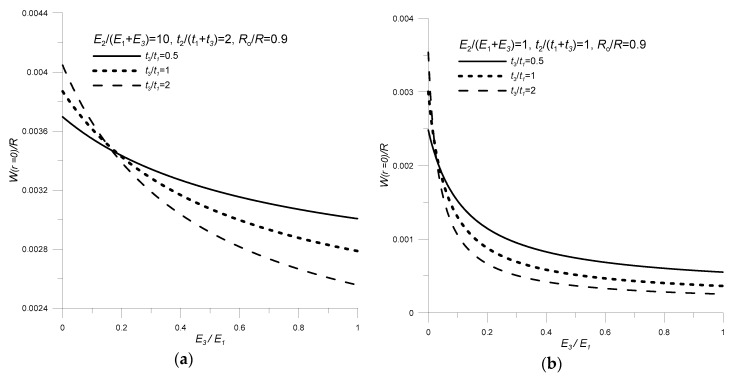
Influence of relative thickness and the ratio of elastic moduli of non-electrical materials on transducer deflection (**a**) *E_g_* = 10, (**b**) *E_g_* = 1; *ν*_1_ = 0.34, *ν*_3_ = 0.46, *R* = 0.06m, *R*_o_/*R* = 0.9, *V* = 100 V.

The results obtained are represented in Figure 8.

It was assumed that the non-piezoelectric ring in the middle layer, made of foam, was used to stabilize the transducer supply cables. Therefore, in the carried-out analyses’ constant parameters −*t*_4_ = *t*_2_, *E*_4_ = 35.8 × 10^6^ Pa, *ν*_4_ = 0.383—were adopted for this material. In addition, it was assumed that the reference material is piezoelectric material PZT-5H, for which, *E*_2_ = 1/*s*_11_ = 6.06 × 10^10^ Pa, *ν*_2_ = −*s*_12_/*s*_11_ = 0.289, *d*_31_ = −2.74 × 10^−10^ m/V. The dimensions and material constants of the other elements were variable and depended on the values of factors (*t_g_*, *E_g_*, *R_g_*, *t_np_*, *E_np_*), which were taken into account when testing the transducer deformation conditions.

Analyzing the results from the first stage, shown in Figure 7, it can be concluded that the transducer deflection increases with the increase in relative thickness (*t_g_*) and stiffness (*E_g_*) of piezo and non-piezoelectric materials. However, the most important role is played by the relative radius of the piezoelectric disk and non-piezoelectric layers—*R_g_*. If the values of this parameter are too small or too large, the transducer’s deflection is very small. In the case where *R_g_* tends to 0 (a piezoelectric element with a small radius *R_o_*), there is a small elongation of the piezoelectric element, and therefore a small deflection of the transducer. In the opposite situation, when the radius of the piezoelectric element is similar to the radius on which the transducer is mounted (*R_g_* tends to 1), the elongation of the piezoelectric element is blocked by forces occurring in the mounting place, which results in a decrease in transducer deflection.

With regard to the mutual geometrical–material relations of non-piezoelectric layers and their impact on transducer deflection, it can be concluded (Figure 8) that:

- the transducer deflection increases as the rigidity of one of the non-electrical components decreases (*E_np_* decrease);
- an increase in the relative thickness *t_np_*, depending on the ratio of elastic moduli *E_np_*, may cause an increase or decrease in the transducer deflection value.

As mentioned in Section 2.1, the analytical model omits the influence of the longitudinal and shear piezoelectric effect on transducer deformation. Such simplification, in some situations, may cause that the deflection determined with the analytical solution may differ from the actual deflection of the transducer. Both geometric and material parameters can influence the magnitude of error. Studies on two-layer circular transducers have shown that the results obtained through the analytical solution when using specific elastic moduli ratio [21] or relative thickness [39] of piezo and non-piezoelectric components differ significantly from the experimental results. Therefore, it is planned to perform additional experimental and numerical tests that will allow determining the degree of applicability of the obtained analytical solution. 

## 5. Summary and Conclusions

In summary, a novel piezoelectric transducer is presented in this work, for which a new analytical model describing its coupled mechanical and electrical properties (a static electromechanical characteristic) has been developed. The transducer is made of two passive plates forming its outer layers. Between the passive plates, there is a piezoelectric disk and a ring made of non-piezoelectric material (e.g., foam). This design protects the electrical components of the converter against harmful external factors (e.g., high-temperature, chemically active atmosphere). Moreover, if sparking occurs on the transducer wires or electrodes, the outer layers will prevent fire. Such functional features of the transducer may allow its use, e.g., as an actuator of the fuel injector control valve in petrol/gas-powered engines [40], or as an alternative drive (instead of magnetic circuit) for regulators used in anti-lock braking systems (ABS) [41].

The obtained analytical model, developed based on the Plates Theory and constitutive equations of piezoelectric materials, allows the prediction of deformations of two- and three-layer transducers with arbitrarily defined geometrical and material parameters. Furthermore, this model was successfully verified by comparing the obtained electromechanical characteristics with those available in the literature [24,25] and with the characteristics achieved through FEM. 

The effect of selected non-dimensional variables on the novel transducer performance has also been examined. These parameters include its dimensions (both relative thickness and relative radii of piezo and non-piezoelectric components) and mechanical properties (elastic moduli ratio of passive and piezoelectric plates), which strongly influence the behavior of the transducer.

Both the developed analytical model (obtained as the closed-form analytical equations) and the analyses performed are important in the process of designing two- and three-layer piezoelectric transducers. Engineers may use them to predict deflection and to optimize converter performance. The results presented in this paper can also be used by other scholars as comparative data.

Future work may be focused on obtaining an analytical solution, taking into account the presence of adhesive layers between the transducer components. Experimental studies to ultimately verify theoretical works are also planned. The application of the analyzed sensor is also envisaged, for which the design parameters will be optimized based on the analytical model, in the fuel injector of a gas-powered engine.
